# Effect of concentrated growth factors on frequency of alveolar Osteitis following partially-erupted mandibular third molar surgery: a randomized controlled clinical study

**DOI:** 10.1186/s12903-020-01210-7

**Published:** 2020-08-17

**Authors:** Banu Özveri Koyuncu, Gözde Işık, Meltem Özden Yüce, Sevtap Günbay, Tayfun Günbay

**Affiliations:** Department of Oral Surgery, Faculty of Dentistry, Ege Univeristy, Bornova, İzmir, Turkey

**Keywords:** Third molar surgery, Alveolar osteitis, Platelet concentrates, Concentrated growth factors

## Abstract

**Background:**

The aim of this prospective study was to assess the effectiveness of concentrated growth factors (CGF) in preventing the development of alveolar osteitis (AO) after the extraction of partially-erupted mandibular third molars.

**Methods:**

Seventy patients (26 men and 44 women) 18 years or older (mean age 25.86; range 18–35) underwent 140 third molar extractions. All the patients presented with bilateral, partially-erupted mandibular third molars and underwent surgical extractions. In each case, one socket received CGF and the other served as a control. The predictor variable was the CGF application and the sides were categorized as ‘CGF’ and ‘non-CGF’. The outcome variable was the development of AO during the first postoperative week. Other study variables included age and gender. Data were analyzed using Cochran’s Q test with the significance level set at a *P* value less than 0.05.

**Results:**

The overall frequency of AO was 11.4% for the control group. The frequency of AO in the CGF group was significantly lower than in the non-CGF group (*p* < 0.001).

**Conclusions:**

Based on the results of this study, application of CGF fibrin gel may decrease the risk of AO development after mandibular third molar surgery.

**Trial registration:**

This study was registered in ClinicalTrials.gov database on November 1, 2019 (ID: NCT04151147, retrospectively registered).

## Background

Surgical removal of mandibular third molar is one of the most frequent procedures in oral surgery and is associated with various complications, including alveolar osteitis (AO). The incidence of AO has been reported in the literature to be around 3% for all extractions but the incidence rises to 30% for cases following surgical extraction of mandibular third molars [1–3]. Several risk factors for AO development have been identified, such as preoperative infections (pericoronitis, periodontal disease, odontogenic abscess, etc.), poor oral hygiene, smoking habits, alcohol usage, menstrual cycle in woman, use of oral contraceptives, surgeon experience, difficulty of surgery, operation time, amount of trauma and socket irrigation [2–4]. As a factor, the relevance of the connection between the development of AO and patient’s age or gender is not evident in the literature, but some studies reported that AO prevalence is slightly higher after the second decade of life, especially for female patients [3, 5–7].

Many attempts have been made to reduce this postoperative complication, which can significantly affect patient’s quality of life in the week following surgery, such as: altered surgical technique (flap designs, different kind of drains) [8–10]; systemic antibiotic use; intra-alveolar application of medicaments or novel products such as chlorhexidine gel, exogenous retinoic acid, honey, herbal extracts and platelet concentrates [11–18].

The initial production of the platelet concentrates began with platelet-rich plasma [19], which was introduced in 1998, and was followed by platelet-rich fibrin PRF [20] in 2000. Then in 2006 *concentrated growth factors* (CGF) was developed by *Sacco* [21]. These products are used to improve the healing process through the release of platelet-derived growth factor (PDGF), transforming growth factors ß-1 (TGF-β1), epidermal growth factor (EGF), fibroblast growth factor (FGF), insulin growth factor-I (IGF-I) and vascular endothelial growth factor (VEGF), which act by stimulating cell proliferation, chemotaxis and angiogenesis [22]. Many studies have shown that use of PRP and PRF in tooth extraction stimulates osseous and soft tissue regeneration, and helps to reduce inflammation, pain, and other side effects [16, 17, 23, 24].

CGF has a dense structure compared to other biomaterials, due to the gradual centrifuge method, and shows higher tensile strength and higher viscosity than early generation platelet concentrates like PRF. The fibrin stucture acts as a scaffolding material and as a reservoir in releasing growth factors at the application site [25–27].

Therefore, the aim of this study was to assess the influence of CGF on AO after surgical removal of partially-erupted mandibular third molars.

More specifically, the purpose of this study was to answer the following question: “In cases of individuals who have partially-erupted mandibular third molars removed, is there a lower incidence of AO in those sides treated with CGF than in the opposite sides not treated with CGF?” In our study, we hypothesized that local application of CGF in lower third molar extraction could reduce the rate of AO.

Our aims therefore were: (1) to estimate the incidence of AO after surgical extraction of partially-erupted mandibular third molars and (2) to compare the incidence of AO in the CGF-treated sockets against the controls which did not received CGF.

## Methods

The investigators designed and implemented a randomized, split-mouth, single-blind clinical trial. The study was conducted at the Ege University, Faculty of Dentistry, Department of Oral Surgery, İzmir, Turkey between January 2018–July 2018. The protocol was approved by the local ethics committee (no:18–4/38) and all participants gave consent for the study. This study was registered at the Clinical Trials (NCT04151147).

### Inclusion criteria

- Patients Age ≥ 18 years of age
- Patients with the need of extraction of mandibular third molars
- Patients willing to participate and able to provide informed consent
- Patients able to cooperate with the requirements of the study protocol
- Healthy patients without medical diseases or a history of bleeding problems
- The third molars had to be symmetrical, partially-erupted and in Class I, Level B, according to the Pell & Gregory classification [28] and in vertical angulations according to Winter’s classification [29].

### Exclusion criteria

- Patients who had pre-existing abscess or cellulitis, acute pericoronitis, or pre-existing conditions such as an odontogenic cyst or tumour associated with their third molars
- Patients who were pregnant and breastfeeding
- Patients who used drugs such as bisphosphonate, steroids and antidepressants
- Patients who had a smoking habit

### Randomization

The participants were evaluated by two examiners (M.Ö.Y and G.I). Randomization was performed by simple coin toss to select the side of CGF fibrin gel placement before the commencement of third molar surgery. Allocation was implemented by a single examiner who was blind to the surgical procedure. In this way, the sides in each patient were randomly divided into 2 study groups (Table 1).

**Table 1:** Study protocol

***Surgical Protocol***	
• Blood collection from patient	
• Elevation of an envelope flap under local anesthesia	
• Bone removal; tooth luxation and extraction; socket irrigation	
CGF group;	
• CGF fibrin gel placement in the extraction socket	
Non-CGF group; • Natural healing after extraction	
• Wound closure on both sides	
• Prescription of antibiotic and anti-inflammatory analgesic	
• Providing postoperative instructions	
***Postoperative Follow-up Protocol***	
• Clinical evaluation on 3rd and 7th days	
• In the case of AO development; Socket curretage, intra-alveolar dressing	

Group I (test) – with CGF placed in the extraction socket.

Group II (control) – without CGF placement.

All the selected patients underwent bilateral surgical extraction of partially-erupted third molars in a single appointment. Following surgery, the surgeon was informed as to which side the CGF was placed. On one side, after removal of the partially-erupted mandibular third molar, CGF fibrin gel was placed, and the socket was sutured (test side). On the other side, after the extraction, the socket was sutured only (control side).

The patients were blind to the side in which CGF fibrin gel had been inserted while the surgeon was not blind to the CGF application or the suturing. For this reason, the clinical evaluation was performed by a second person who was blind to the side into which the CGF fibrin gel was placed (G.I).

### Study variables

The primary predictor variable was the application of CGF fibrin gel.

#### CGF preparation

CGF was prepared according to Sacco [21] protocol. The patients’ blood was collected in two sterile, disposable, silica-coated, 9 ml glass tubes with vacutainers. The tubes were centrifuged immediately to prevent coagulation in a specialized centrifuge device (Medifuge, France). The CGF program was set up and the centrifuge was programmed with the following data: accelerated for 30 s so as to reach 2700 rpm, rotated for 2 min, then reduced to 2400 rpm, then rotated again for 4 min and accelerated to 2700 rpm, rotated for 4 min, then accelerated to 3000 rpm for 3 min, and decelerated for 36 s to stop. Three layers were observed in the tubes: the upper layer with platelet-poor plasma and the lower layer with red blood cells, separated by the “buffy coat” containing the CGF fibrin gel using forceps and scissors .

The primary outcome variable was AO, classified as present or absent.

Clinical data was collected with regard to AO formation on the 3rd and 7th days after surgery. Symptoms of this postoperative complication were evaluated with the following characteristics: (1) postoperative pain with increasing severity 2 to 3 days in, and around the extraction site; (2) partial or total loss of blood clot and exposure of the alveolar bone with or without halitosis [30, 31]. Other defining symptoms such as radiating pain towards the temporal region and ear, inflamed gingival margin, ipsilateral regional lymphadenopathy and, less commonly, low-grade fever, were also noted [13, 31, 32].

Patients who developed AO were treated in accordance with the clinical protocols that have been reported in the literature [3, 5]. Treatment of AO was performed by the same blinded examiner. Curettage was performed to form the fibrin clot, and an intra-alveolar dressing with eugenol (Alveogyl®, Septodont, Kent, England) was placed into the non-healing sockets.

Data was also collected with regard to demographic variables (age, gender), preoperative variables (extraction difficulty, surgeon experience), and perioperative variables (volume of irrigation, duration of surgery).

All operations were performed under local anesthesia by the same surgeon (B.Ö.K). The surgeon who had operated on the patients was not involved in either the preoperative or the postoperative assessment. For the inferior alveolar block 2% lidocaine hydrochloride 2 ml with 1:80.000 epinephrine was used (Jetokain®, Adeka, İstanbul, Turkey). An envelope flap was raised to provide access.

Bone removal was carried out with the aid of stainless steel burs. A straight handpiece and micromotor were used. Constant irrigation with saline was applied while removing the bone, to prevent thermal necrosis. The third molar was luxated with the help of a straight elevator and then extracted using third molar forceps. After extraction, any remains of the dental follicle were removed and the extraction sockets were irrigated with 60 mL of sterile saline. To prevent laceration of the flap, bone contouring was also performed under sterile saline irrigation. CGF fibrin gel was then randomly placed into one socket and the opposite side was used as the control. Finally, wound closure was completed with silk suture (4/0 silk, ½ cutting edge, 75 cm, Doğsan, İstanbul, Turkey). All patients were given amoxicillin+clavulanic acid (625 mg/12 h, Augmentin-BID®, Glaxo-Smith Kline, United Kingdom) and naproxen sodium (550 mg/12 h, Apranax Forte®, Abdi İbrahim, İstanbul, Turkey) for a period of 7 days after surgery. Postoperative instructions were given to the patients. Each patient returned for clinical evaluation on the third and seventh days after surgery to evaluate AO. Sutures were removed 7 days after surgery.

### Sample size calculation

Sample size was calculated with PASS 2000 software [33]. An incidence of 10% in the CGF group and 30% in the control group was estimated to detect a difference (P0 - P1) with the binomial hypothesis test [31, 34]. This showed that 57 subjects would be sufficient to obtain 96% power in detecting a statistical difference between the test and control groups, with a target significance level of 0.05.

### Data analysis

The Cochran’s Q test was used to determine whether there was a difference in primary outcome AO among the 4 related samples (CGF group: third and seventh days and control group: third and seventh days). After a significant Cochran’s Q test result, Dunn’s test with Bonferroni correction was applied for pairwise comparisons. Categorical data were described using observed frequencies and percentages, and continuous variables were summarized by their means and standard deviations. In the study, *p* values < 0.05 were considered to be statistically significant and IBM SPSS software was used for all statistical analysis (IBM Corp. Released 2017. IBM SPSS Statistics for Windows, Version 25.0. Armonk, NY: IBM Corp.)

## Results

**Fig. 1:**
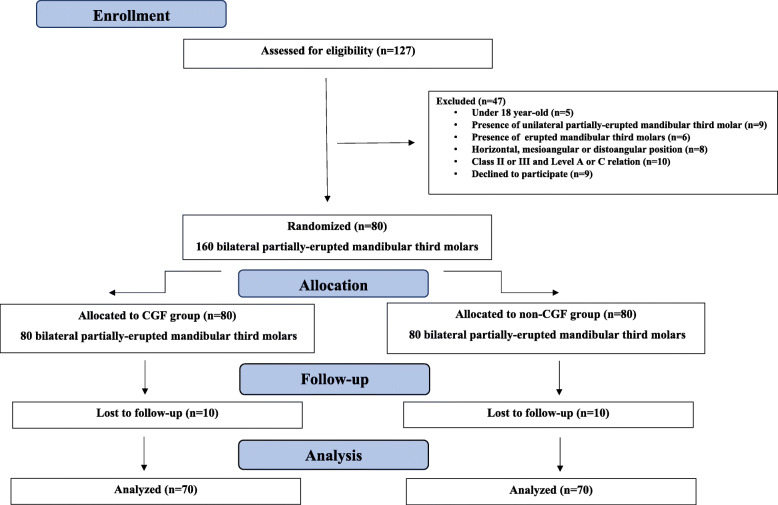
Study Protocol

Eighty patients (50 female and 30 male, aged 18–35, mean age: 27.9 years) met the inclusion criteria and were initially entered into the study. However, subsequently excluded from this number were 10 patients who did not return for evaluation: three patients did not arrive for the postoperative third day control; three did not allow AO treatment; four patients did not arrive for the postoperative seventh day control. The remaining 70 patients (44 female and 26 male, age range 18–35 years, mean age: 25.86 years) completed the study (Fig. 1). No surgical complications were recorded during tooth extraction. Demographic variables, including sample size, age and gender, were evaluated and no significant differences were observed between the two groups (Table 2). All operations were performed by the same surgeon, no differences were reported in the amount of irrigation or the duration of surgery (a mean score of 11.68 min for the CGF group and 11.40 min for the control group) as perioperative variables.

**Table 2:** Demographic variables and AO frequency

Study variable	CGF Group	Control Group	*p* value
Demographic variables:			
Sample size (n)	70	70	N/A
Age, yr	25.86 ± 4.42	25.86 ± 4.42	0.98
Sex:male, n	26	26	0.12

Values are presented as mean ± SD or n

*n* No. of patients, *N/A* not applicable;

Seventy patients were examined to determine whether the incidence of AO had changed over time and over the study groups. According to the Cochran’s Q test result, there was a significant difference among the four related proportions (χ2(3) = 24, *p* < 0.001). The incidence of AO in the control group (*n* = 8; 11.4%) was found to be higher than the test group (n = Ø), and the difference between the study groups was statistically significant at the third day, postoperatively (*p* < 0.001). The incidence of AO was significantly different for the control group between the third and seventh days (*p* < 0.001). Healing was uneventful in the control group following treatment of AO and there was no statistically significant difference between the two groups on the postoperative seventh day (*p* = 1.000; Table 3).

**Table 3:** Primary predictor variable versus the primary outcome variable, alveolar osteitis (AO)

Day	CGF-AO		NonCGF-AO	
	AO present (n %)	AO absent (n %)	AO present (n %)	AO absent (n %)
3^a^	0 (0%)	70 (100%)	8 (11.4%)	62 (88.6%)
7^a^	0 (0%)	70 (100%)	0 (0%)	70 (100%)
**p value**^a^	***p*** **< 0.001**			
**Differences**	**Pairwise Comparisons**^**b**^			
CGF-NonCGF at 3rd day	*p* < 0.001			
CGF-NonCGF at 7th day	1.00			
3rd-7th days in CGF	1.00			
3rd-7th days in NonCGF	*p* < 0.001			

*Abbreviations*: *AO* alveolar osteitis, *CGF* concentrated growth factors

^a^Cochran’s Q test Test χ2(3) = 24

^b^With Bonferroni correction

## Discussion

The purpose of this study was to answer the question of whether the local application of CGF reduced the incidence of AO when compared with a control group not treated with CGF. Our hypothesis was that the local application of CGF in lower third molar extraction could reduce the rate of AO. The results revealed that the frequency of AO in the CGF group was significantly lower than in the non-CGF group. Our hypothesis therefore was accepted.

AO is one of the most common complications following third molar surgery and also one of the most studied complications in dentistry. Therefore, it is key to the success of the procedure [3, 5]. Although there have been many attempts by clinicians to reduce the incidence of AO using novel methods and products, there is still debate regarding the most effective method [11–18].

Platelet concentrates for medical and dental use are autogenous regenerative preparations obtained through the centrifugation of a blood sample from the patient [35]. Several in vitro studies, animal experiments and clinical studies suggest that platelet concentrates have certain properties which effectively trigger the stimulation of soft tissue regeneration and bone healing, and also act to reduce inflammation, pain and side effects [18, 23, 36–40].

Our search of the literature found that the application of PRP and PRF in the extraction socket produced better results and either prevented or reduced the incidence of dry socket [4, 41, 42]. In a clinical study, Rutkowski et al. [41] evaluated the effect of PRP on the prevention of AO after a total of 904 mandibular extractions. They found that the application of PRP reduced the occurrence of AO by up to 60% in high-risk patients. Similarly, Eshghpour et al. [4], and He et al. [42], reported that the application of PRF after third molar surgery promotes clot formation and reduces mechanical dislodgement. They found that the incidence of AO in the PRF group was significantly lower than in the non-PRF group. Therefore, their conclusion was that the fibrin matrix reduces the incidence of AO.

More randomized clinical studies with larger sample sizes are needed to understand the effects on AO occurrence following lower third molar surgery [40, 43]. The preventive qualities of AO act to prohibit bacterial formation and to control bleeding until the socket is healed [44]. From the literature on antimicrobial properties of platelet-rich preparations, PRP and PRF seem to inhibit bacterial growth during the first hours of incubation. However these concentrates are unlikely to be capable of breaking down the microbial load completely [35]. Moreover, abundant growth factors in platelet concentrates are signaling molecules which lead to hematopoiesis and wound healing in the early phase [45]. However, platelet concentrates differ in their ability to release of growth factors. PRP secretes more than 95% of presynthesized growth factors within 1 h [46] while PRF can continue releasing growth factors for at least 1 week [47]. Therefore, treatment outcomes at the application site are difficult to predict.

Our research has focused on the effect of the application of CGF to AO, because of the acknowledged superior performance of CGF compared to PRP and PRF.

CGF, the second-generation platelet concentrate which was developed by Sacco [21] in 2006, contains more growth factors and has a denser structure than PRP and PRF [25]. The concept of CGF has been examined and reported in several studies in the literature relating to tissue healing [36–39].

Masuki et al. [36] demonstrated the high level of growth factors, including PDGF, TGF-β1, VEGF and pro-inflammatory cytokines, in CGF. They found that both PRF and CGF preparations contain significant amounts of growth factor capable of stimulating periosteal cell proliferation. Also, Takeda et al.’s [37] preliminary results showed that fibrin and soluble factors in CGF stimulated initial cell stretching, proliferation, and osteoblastic differentiation of RBM cells in vitro. They also had a similar effect on bone regeneration in rat calvarial bone defects in vivo.

In a clinical study, Tanaka et al. [38] reported that CGF clots help wound healing, particularly in the case of diabetic patients affected by problems in healing due to microangiopathia.

In our survey of the literature, we found no mention of the role of CGF in the prevention of AO. Therefore it appears that this study is the first to examine the effect in the context of the removal of partially-erupted third molars.

Our study showed that the application of CGF significantly reduced the incidence of AO in the test group compared with the control. This indicated that CGF has a potential benefit in the first phase of healing. However, further randomized studies with a large sample are necessary to confirm these preliminary findings.

Moreover, the absence of AO in the test group may dependent on the biological behaviour of CGF. Sohn et al. [48] reported that the CGF matrix dissolved and remodeled slowly following application, in a similar manner to a natural blood clot, and that it compared well to PRP. In this way, CGF prolongs the duration of growth factor activity, which is conductive for growth factor synergy, enhancing cell proliferation and osteogenic differentiation [35]. Similarly, Qin et al. [49] reported that CGF could release TGF-β1, which has a potential role in wound healing, for a period of 13 days at least. From the previous information, it can be concluded that CGF matrix dissolved slowly and acted as a scaffold in the extraction sockets of the CGF group, and this may have helped to prevent the occurence of AO.

One risk factor in the development of AO is the degree of surgical trauma during third molar surgery, as trauma can release tissue activators [50]. The extent of the surgeon’s professional experience is generally a factor in the degree of surgical trauma experienced by the patient [4, 51]. This variable was eliminated by having a single experienced surgeon perform all the extractions in this study.

In addition, the amount of bone removal and the position of the tooth [52, 53] affect the degree of surgical trauma. This study was designed as a split-mouth model, in which each partially-erupted third molar had a contralateral tooth with the same difficulty level. Efforts were made to minimize the possible influence of difficulty level as a confounding factor in the development of AO, and in the evaluation of the CGF effect.

Irrigation of the surgical site could be another risk factor in AO frequency [54]. Butler and Sweet [55] reported that 60 ml of irrigation is required in order to reduce the risk of AO development, as a large irrigation volume is needed to effectively remove contaminants such as debris and bacteria. With regard to our own findings, no differences were noted in the amount of irrigation between the groups. To eliminate this risk factor, all the extraction sockets were irrigated using 60 ml of sterile saline.

It is necessary to highlight some limitations in our study: The allocation was implemented before third molar extractions, in contrast to cases in the literature [31]. This could give rise to some bias in the study. Also, the effect of CGF on the periodontal health of the second molar was not evaluated in this study, because of short-term follow up. Further randomized studies are required with a large sample group of patients with bilateral impacted third molar who are at high risk of severe swelling, pain, and trismus, and also to investigate the effect of CGF on the periodontal health of the second molar following third molar surgery, with a longer follow-up.

## Conclusions

The procedure of CGF preparation is simple and cost effective. This clinical study shows that the application of CGF had a significant effect on AO after partially-erupted mandibular third molar surgery and may be recommended to reduce the risk of developing AO following third molar extraction. However, further clinical studies will be required with a larger sample group in order to confirm these preliminary findings.

## Data Availability

The datasets used and/or analysed during the study are available from the corresponding author on reasonable request.

## Abbreviations

CGF: Concentrated growth factors
AO: Alveolar osteitis
PRF: Platelet rich fibrin
PRP: Platelet rich plasma
PDGF: Platelet-derived growth factor
TGF- β1: Transforming growth factors
EGF: Epidermal growth factor
FGF: Fibroblast growth factor
IGF-I: Insulin growth factor-I
VEGF: Vascular endothelial growth factors
RPM: Rounds per minute
RBM: Red blood mononuclear cells
